# Spectrophotometric and Chromatographic Assessment of Total Polyphenol and Flavonoid Content in *Rhododendron tomentosum* Extracts and Their Antioxidant and Antimicrobial Activity

**DOI:** 10.3390/molecules29051095

**Published:** 2024-02-29

**Authors:** Halyna Kukhtenko, Nataliia Bevz, Yulian Konechnyi, Oleksandr Kukhtenko, Izabela Jasicka-Misiak

**Affiliations:** 1 Institute of Chemistry, University of Opole, 48 Oleska Str., 45-052 Opole, Poland; izajm@uni.opole.pl; 2 Department of Cosmetology and Aromology, National University of Pharmacy, 53 Pushkinska Str., 61002 Kharkiv, Ukraine; 3 Department of Pharmaceutical Chemistry, National University of Pharmacy, 53 Pushkinska Str., 61002 Kharkiv, Ukraine; nata.bevz.60@gmail.com; 4 Department of Microbiology, Danylo Halytsky Lviv National Medical University, 69 Pekarska, 79010 Lviv, Ukraine; yuliankonechnyi@gmail.com; 5 Department of Technology of Pharmaceutical Preparations, National University of Pharmacy, 53 Pushkinska Str., 61002 Kharkiv, Ukraine; kukhtenk@gmail.com

**Keywords:** *Rhododendron tomentosum*, *Ledum palustre*, rutin, hyperoside, quercetin, chlorogenic acid, aluminum chloride, Folin-Ciocalteu reagent, DPPH assay, UV–Vis, HPTL, antimicrobial activity, antioxidant activity

## Abstract

In the literature, the chemical composition of *Rhododendron tomentosum* is mainly represented by the study of isoprenoid compounds of essential oil. In contrast, the study of the content of flavonoids will contribute to the expansion of pharmacological action and the use of the medicinal plant for medical purposes. The paper deals with the technology of extracts from *Rh. tomentosum* shoots using ethanol of various concentrations and purified water as an extractant. Extracts from *Rh. tomentosum* were obtained by a modified method that combined the effects of ultrasound and temperature to maximize the extraction of biologically active substances from the raw material. Using the method of high-performance thin-layer chromatography in a system with solvents ethyl acetate/formic acid/water (15:1:1), the following substances have been separated and identified in all the extracts obtained: rutin, hyperoside, quercetin, and chlorogenic acid. The total polyphenol content (TPC) and total flavonoid content (TFC) were estimated using spectrophotometric methods involving the Folin-Ciocalteu (F-C) reagent and the complexation reaction with aluminum chloride, respectively. A correlation analysis was conducted between antioxidant activity and the polyphenolic substance content. Following the DPPH assay, regression analysis shows that phenolic compounds contribute to about 80% (*r*^2^ = 0.8028, *p* < 0.05) of radical scavenging properties in the extract of *Rh. tomentosum*. The extract of *Rh. tomentosum* obtained by ethanol 30% inhibits the growth of test cultures of microorganisms in 1:1 and 1:2 dilutions of the clinical strains #211 *Staphylococcus aureus* and #222 *Enterococcus* spp. and the reference strain *Pseudomonas aeruginosa* ATCC 10145.

## 1. Introduction

*Rhododendron tomentosum* (formerly *Ledum palustre*) is an evergreen, squat shrub that has a significant distribution area on the territory of Ukraine throughout its northern part [1]. *Rh. tomentosum* is widespread mainly in wet and swampy coniferous and deciduous forests, sphagnum marshes, and peatlands in the northern regions of Europe, North America, including the northern parts of Canada and Alaska, Siberia, and North Asia [2]. The popular names of *Rh. tomentosum* are marsh labrador tea, northern labrador tea, and wild rosemary [3,4,5]. As a raw material of *Rh. tomentosum*, only young shoots of the first year up to 10.0 cm long are harvested. They are crushed together with flowers and leaves. The *Rh. tomentosum* has a low level of regeneration, so after cutting the shoots, the biomass is restored only after 3 years. *Rh. tomentosum* is a pharmacopeial plant raw material in France and Germany (French Pharmacopoeia, 2007; German Homoeopathic Pharmacopoeia, 2000). 

In traditional Ukrainian medicine, the shoots of *Rh. Tomentosum* are used in the form of an aqueous infusion or decoction. Historically, this infusion has been used to treat various respiratory and pulmonary ailments, such as bronchitis, tuberculosis, pertussis (whooping cough), and asthma. On the other hand, decoction and oil infusion are used as an ointment formulated with an animal fat base, which is applied topically to address issues such as eczema, scabies, and insect stings. And the shoots of *Rh. Tomentosum* have long been used as an insecticide to repel moths and other insects by placing the shoots indoors. In the pharmaceutical market of Ukraine, there is a plant blend containing the shoots of *Rh. Tomentosum*, “Fitobronkhol” (Liktravy, Zhytomyr), and separately, the shoots of *Rh. tomentosum* (PJSC Pharmaceutical Factory “VIOLA”, Zaporizhzhia) can be purchased at a pharmacy to treat diseases that are accompanied by cough in the form of an infusion (10 g in 200 mL of water at the dose of ¼ of a glass, 2–3 times per day).

In the traditional medicine of other peoples of the world, *Rh. tomentosum* has been used to treat headaches, toothache, pain, and shingles. The leaves are also used as marsh tea, which is considered to act as an abortifacient, diaphoretic, diuretic, emetic, expectorant, and galactagogue. *Rh. tomentosum* leaves and shoots are used in China to treat coughs and asthma, to decrease blood pressure, and as an antifungal agent [6,7,8]. 

Despite the long tradition of its use, this plant has always been treated with caution due to its essential oil content. The fragrance of *Rh. tomentosum* is so intense that it can cause a headache. Drinking excessive amounts of *Rh. tomentosum* tea is not recommended, as it is very strong and can lead to serious side effects, including intestinal disturbances, drowsiness, and a strong diuretic effect. *Rh. tomentosum* has a high content of volatile compounds in the essential oil; one of the more toxic compounds involved is the sesquiterpenoid ledol, which has an effect on the nervous system [6].

Table 1 shows collected information on the results of the study of the pharmacological activity of shoots, which are presented in scientific publications and placed in publicly accessible reference databases. 

*Rh. tomentosum* is considered by scientists primarily as an ethereal medicinal plant, but it also contains other groups of biologically active substances, such as phenol carboxylic acids, flavonoids, polysaccharides, and pectin substances [5,8]. The chemical composition of the plant in the literature is represented mainly by the study of isoprenoid compounds of essential oil. In contrast, the content of polyphenolic compounds has not been studied enough. Given the wide biological activity of polyphenolic compounds as flavonoids, it is important to study the technology of extracts from the shoots of *Rh. tomentosum* and carry out a qualitative and quantitative study of their composition [26]. Thus, extraction of dried plant material with different solvents was performed, and characterization of the obtained extracts was achieved by chromatographic (HPTLC), spectrophotometric, and microbiological methods. 

## 2. Results

For the research, 10 samples of extracts of *Rh. tomentosum* were prepared, nine of which were obtained by extraction with ethanol of various concentrations from 10% to 90% in steps of 10% (EtOH10, EtOH20, etc.) and one with purified water (EtOH0). On the one hand, this approach was chosen due to the fact that ethanol is the most acceptable compound in pharmaceutical drug technology due to its relatively safe toxic profile, and on the other hand, the studied group of biologically active substances, polyphenolic compounds, are soluble in a wide range of ethanol concentrations [27,28,29]. The liquid extracts were obtained by the method of threefold fractional maceration in combination with the effect of ultrasound in the ratio of raw materials/extractant 1:20. The first portion of the extract was collected separately, the second and third portions were combined and evaporated to dryness, and the resulting dry residue was dissolved in the first portion of the extract. Thus, the extracts to be studied were prepared in the ratio of raw material to extractant 1:5. 

### 2.1. HPTLC-UV/Vis Method Development

**Figure 1:**
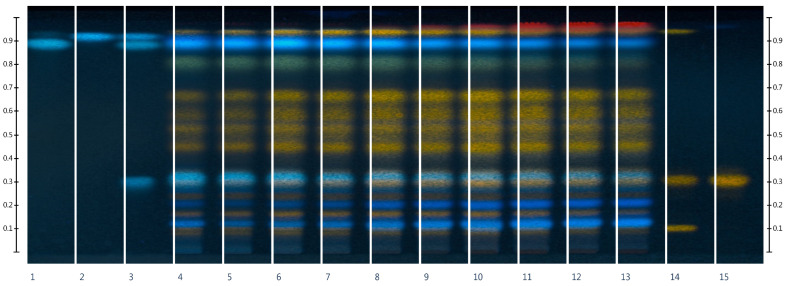
HPTLC fingerprints of the extracts of *Rh. tomentosum* and standards with increasing Rf at λ 366 nm after derivatization: 1—rosmarinic acid, 2—caffeic acid, 3—chlorogenic acid + rosmarinic acid + caffeic acid, 4—EtOH0, 5—EtOH10, 6—EtOH20, 7—EtOH30, 8—EtOH40, 9—EtOH50, 10—EtOH60, 11—EtOH70, 12—EtOH80, 13—EtOH90, 14—rutin + hyperoside + quercetin, 15—hyperoside.

**Figure 2:**
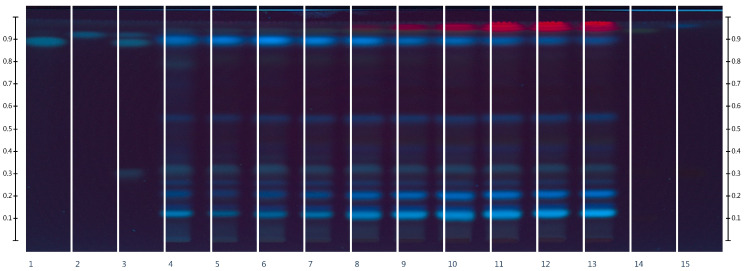
HPTLC fingerprints of the extracts of *Rh. tomentosum* and standards with increasing Rf at λ 254 nm before derivatization: 1—rosmarinic acid, 2—caffeic acid, 3—chlorogenic acid + rosmarinic acid + caffeic acid, 4—EtOH0, 5—EtOH10, 6—EtOH20, 7—EtOH30, 8—EtOH40, 9—EtOH50, 10—EtOH60, 11—EtOH70, 12—EtOH80, 13—EtOH90, 14—rutin + hyperoside + quercetin, 15—hyperoside.

The purpose of the chromatographic study was to identify flavonoids in *Rh. tomentosum* using active markers, which were subsequently used as standard samples for the quantitative determination of the total flavonoid content by the UV–Vis method. For the chromatographic separation of polyphenol compounds, several eluent systems were tested: ethyl acetate/methanol/water/formic acid (50:10:7:1), ethyl acetate/methyl ethyl ketone/formic acid/water (50:30:10:10), and ethyl acetate/water/formic acid/acetic acid (68:18:7:7). The highest resolution of flavonoids and phenolic acids was obtained using the solvent system ethyl acetate/formic acid/water (15:1:1); the obtained HPTLC chromatograms are shown in Figure 1 and Figure 2. 

In the process of HPTLC fingerprint analysis, the identification of substances is based on the Rf value (position of the bands and color background) of the standard substances and the studied extracts [30,31]. HPTLC chromatograms visually show similarities and differences in the composition of the test samples. As can be seen from the results, on the chromatographic strips of the test samples scanned after derivatization in UV light at 366 nm, there are zones that correspond in color and Rf to the standard samples of rutin Rf (0.12), hyperoside Rf (0.32), and quercetin Rf (0.94) [32,33]. As can be seen from Figure 1, rutin, hyperoside, and quercetin are present in all extract samples regardless of the extractant, but in terms of the intensity of color and the size of the colored zones, it can be argued that samples in which the extractants were purified water and ethanol 10% are somewhat inferior. In addition to flavonoids, standards of phenolic acids, namely chlorogenic acid, rosmarinic acid, and caffeic acid, were applied to the chromatographic plate [34,35]. 

After scanning chromatographic zones before derivatization in UV light at 254 nm (Figure 2) and after detection in UV light at 366 nm (Figure 1), chlorogenic acid Rf (0.32) was identified. The substances rutin, hyperoside, quercetin, and chlorogenic acid were also detected by the authors [3,36] in the raw material of *Rh. tomentosum*. The zones of rosmarinic acid and caffeic acid standard substances differ in color in UV light at 254 nm, which may indicate their absence in the raw material.

### 2.2. Determination of Total Phenolic Content

**Figure 3:**
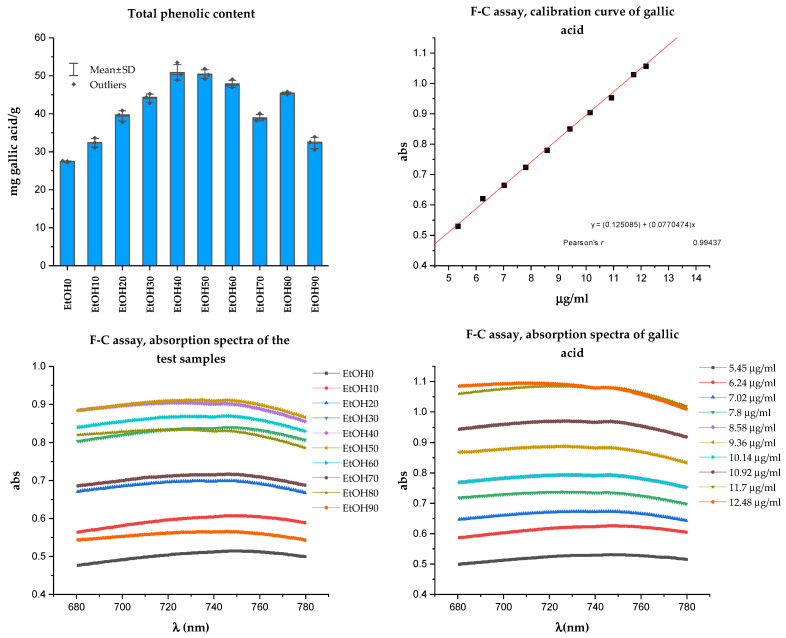
Result of Folin-Ciocalteu assay.

The Folin-Ciocalteu (F-C) assay is a colorimetric method based on single-electron transfer reactions between the F-C reagent and phenolic compounds. Phenolic compounds act as effective oxygen radical scavengers because phenolic radicals’ electron reduction potential is lower than oxygen radicals, and phenoxyl radicals are less reactive than oxygen radicals. Thus, phenolic compounds’ scavenging of reactive oxygen radicals halts further oxidative reactions [37]. The results of determining the total phenolic compounds in the *Rh. tomentosum* extracts are presented in Figure 3. The total phenolic content was expressed as gallic acid equivalents (mg of GAE/g). 

### 2.3. Determination of Total Flavonoid Content

**Figure 4:**
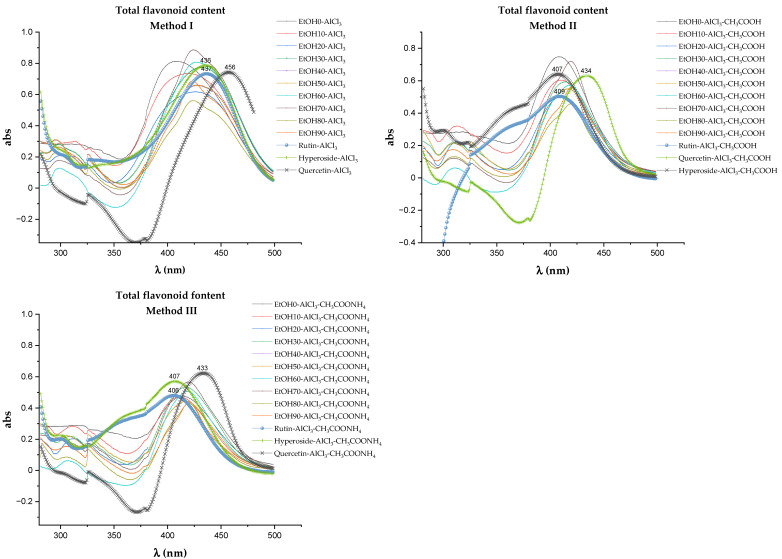
Absorption spectra of the test samples and aluminum chloride complexes.

The most commonly used method for quantifying the total flavonoid content (TFC) is the method of differential spectrophotometry after the complexation reaction with AlCl_3_ [38,39]. Due to the multifactorial dependence of the results of spectrophotometric quantification of the TFC, the reproducibility of the technique receives special attention. Therefore, at the stage of the methodology development, the electronic absorption spectra of extracts and the spectra obtained by three methods that differed in conditions were taken (Figure 4). All samples were tested under the same sample preparation conditions: a reaction time of 30 min at room temperature without direct sunlight [40]. 

**Figure 5:**
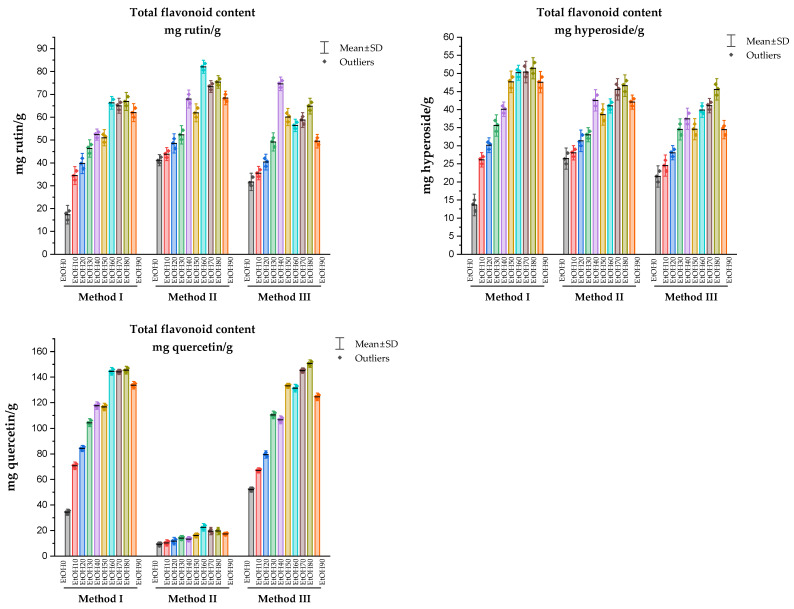
The total content of flavonoids of *Rh. tomentosum* extracts determined using three methods, expressed as equivalents of rutin, hyperoside, and quercetin.

The study of the TFC in the analyzed samples, depending on the extractant used, was carried out in comparison with such standard samples as rutin, hyperoside, and quercetin. The TFC of *Rh. tomentosum* determined in extracts under the conditions of three methods and expressed as rutin, hyperoside, and quercetin equivalents are shown in Figure 5.

### 2.4. DPPH Radical Scavenging Activity

**Figure 6:**
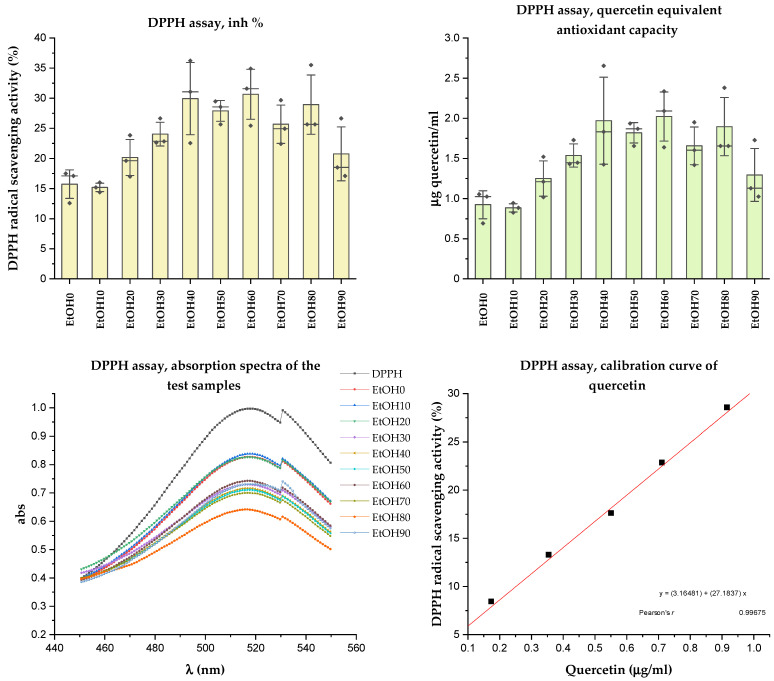
Result of DPPH assay.

The DPPH radical is known for its remarkable stability due to the delocalization of the radical in aromatic rings. The radical is neutralized in assays by accepting either a hydrogen atom or an electron from an antioxidant species (or reducing agent). It is converted into a reduced form (DPPH or DPPH-H) during this process. The unpaired electron of the DPPH radical absorbs strongly at 517 nm, resulting in a deep purple color. However, when the odd electron pairs up with another electron, the initial color gradually fades to pale yellow [37]. The results of radical scavenging activity of the *Rh. tomentosum* extracts are presented in Figure 6. The antioxidant activity was expressed as quercetin equivalent antioxidant capacity (mg QEAC/mL).

### 2.5. Determining Antimicrobial Activity In Vitro

**Figure 7:**
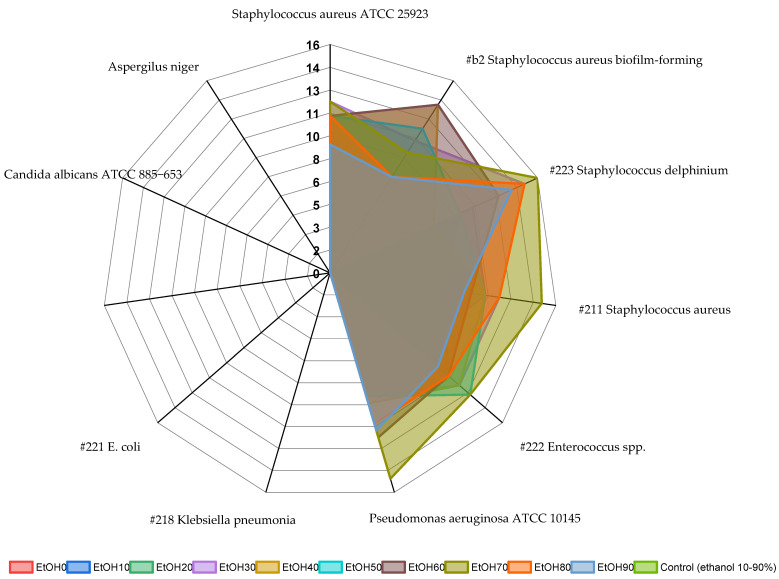
Antimicrobial activity of *Rh. tomentosum* extracts determined by the well diffusion method, shown as diameter of growth inhibition. Extracts EtOH70 and EtOH60 showed the best antimicrobial activity against staphylococci and the reference *Pseudomonas*. Color area = size of growth retardation zone.

By the well agar diffusion method, there were no growth inhibition zones of the tested microorganisms around the wells with the water extract. However, when the extract was dripped onto the inoculated agar, there was growth inhibition. It could be explained by the slow diffusion of the extract into the agar. Ethanol extracts inhibited the growth of Gram-positive bacteria such as staphylococci (#223, #221, and #b2) and enterococci and did not inhibit the growth of Gram-negative microorganisms and fungi (Figure 7). Extracts EtOH70 and EtOH60 showed the best antimicrobial activity against staphylococci and the reference *Pseudomonas*. Despite this, EtOH30-90 extracts inhibited the growth of *Pseudomonas aeruginosa*. It was often possible to observe the secondary growth of microorganisms in the zone of growth retardation of the culture, which indicates an insufficient concentration of the active substance for a bactericidal effect on some microorganisms. Ethanol control in all dilutions did not inhibit the growth of the tested microorganisms.

The water extract EtOH0 partially inhibited the growth of all tested microorganisms in the first dilution (1:1). In the second dilution (1:2), there was no inhibition. A bactericidal effect was observed against *Enterococcus* spp. in the first dilution (the colonies were pale and small in contrast to the colonies from the second dilution) (Table 2). 

Ethanol extracts EtOH10 and EtOH20 inhibit the growth of bacteria in dilutions 1:1 and partially in 1:2. The EtOH10 extract practically did not inhibit the clinical strain of biofilm-forming *Staphylococcus aureus* #b2 and the museum strain of *Pseudomonas aeruginosa* ATCC 10145, while the EtOH20 extract inhibited these strains at 1:1 dilution. Ethanol extract EtOH30 completely inhibits the growth of test cultures of microorganisms in 1:1 and 1:2 dilutions of the clinical strains #211 *Staphylococcus aureus* and #222 *Enterococcus* spp. and the reference strain *Pseudomonas aeruginosa* ATCC 10145. Ethanol extracts from EtOH40 to EtOH90 completely suppress the growth of test cultures of microorganisms in 1 and 2 dilutions, as well as ethyl alcohol (control).

## 3. Discussion

Polyphenols are a numerous class of secondary metabolites of plant raw materials, which today has about 10,000 identified individual compounds [41]. They are produced by plants to protect them from abiotic and biotic factors. They can be classified into five different subgroups based on their structure: phenolic acids (which are further subdivided into hydroxybenzoic, and hydroxycinnamic acids), flavonoids (subdivided into flavonols, flavan-3-ols, flavones, flavanones, isoflavones, flavanonols, and anthocyanidins), coumarins, lignans, and stilbenes. Among them, flavonoids are the most abundant in plants [38].

Using the HPTL method (Figure 1 and Figure 2), flavonols such as rutin, hyperoside, quercetin, and one hydroxycinnamic acid, chlorogenic acid, were identified in all studied extracts of *Rh. tomentosum*. The presence of these substances in the raw materials of *Rh. tomentosum* causes a wide spectrum of pharmacotherapeutic action characteristic of these substances. This may partly explain the effectiveness of infusions and decoctions in folk medicine. At the same time, the presence of various groups of biologically active substances in the raw material may lead to a synergistic pharmacotherapeutic effect. Therefore, appropriate methods are employed to assess the total content of some groups of substances.

Several analytical techniques can determine polyphenol content. An alternative method for evaluating the content of total polyphenols in various samples is to use a spectrophotometric method such as Folin-Ciocalteu (F-C) or a method that utilizes an aluminum complexation reaction for total flavonoids. These methods are not specific and are typically used to assess the total content of all polyphenols or some subgroup of polyphenols. The F-C method is based on single-electron transfer reactions between the F-C reagent and phenolic compounds [37]. The TPC was expressed as gallic acid equivalents. As can be seen from Figure 3, all studied extracts of *Rh. tomentosum* have substances of polyphenol structure; however, the maximum amount is observed in extracts extracted with ethanol at a concentration of 40% and 50% (50.91 ± 2.78 and 50.45 ± 1.60 mgGAE/g, respectively, for EtOH40 and EtOH50). The wide range of ethanol concentrations at which the maximum extraction of polyphenolic substances is observed may be due to the fact that, for example, glycoside and aglycone forms of flavonoids have different solubility in ethanol [28]. 

During the determination of TFC using the complexation reaction with aluminum chloride in various reaction media, variations in the values of the bathochromic shift of the electronic absorption spectrum were noted (Figure 4). Quercetin, rutin, and hyperoside were used as flavonoid markers, which were identified in the extracts of *Rh. tomentosum* by the HTPLC method. Correct determination of absorption maxima affects the final result of calculations of TFC (Figure 5). Numerous scientific publications are devoted to the study of the spectrophotometric behavior of flavonoids in reactions with AlCl_3_, which demonstrate that the differential spectra of flavonoids (wavelength and intensity of maximum absorption) depend on the chemical nature of the flavonoids, the stoichiometric ratio of AlCl_3_ molecules and flavonoids, the pH of the medium, the time and conditions (in darkness or daylight) of the reaction, etc. [42,43,44,45,46,47].

**Figure 8:**
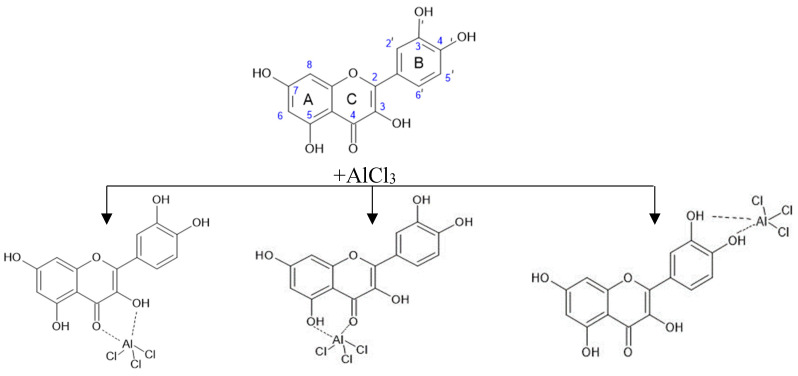
Diagram of possible focal points of binding between the quercetin molecule and AlCl_3_.

According to the scientific literature, the reaction of complexation with AlCl_3_ can potentially have several centers for the course, the localization of which is determined by the presence of a nearby hydroxyl and carbonyl group in the C-4 position of the C-ring [38,39,41,45]. The 3-OH and C=O group (“3-4 site”), 5-OH and C=O group (“5-4 site”), or a pair of 3′-OH and 4′-OH groups of ring B (“3′-4′ site”) can participate in the binding of metal ions. Figure 8 shows possible ways of complexation using the example of the quercetin molecule [38].

A possible simultaneous substitution along the rings A and B leads to a bathochromic shift of bands and an increase in the intensity of absorption [38]. Rutin and hyperoside flavonoid molecules differ from quercetin in the presence of a glycosidic substituent attached to the oxygen atom at position 3, so rutin and hyperoside have a “5-4 site” and a “3′-4′ site” to form complexes with aluminum ions.

The bathochromic shift of absorption maxima in the electronic spectra of both the test samples and the standard substances of rutin, hyperoside, and quercetin compared to the published data in scientific articles [39,43,44] can be explained by the use of ammonium acetate instead of potassium or sodium acetates (Method III) and a different, twice greater concentration of AlCl_3_ in all three methods. 

Under the conditions of an increase in the concentration of AlCl_3_ compared to those described in the literature [44], a significant bathochromic shift is observed as a possible result of interaction at several binding coordination centers in the flavonoid molecule. According to the authors [46], the interaction of flavonoid molecules with aluminum ions occurs sequentially in the order of binding “3′-4′ site”—“3-4 site”—“5-4 site” [46,48]. This means that the binding of aluminum ions to the site “3-4 site” will take place when all “3′-4′ sites” are occupied.

Analysis of the obtained data (Figure 4) indicates that the introduction of donor substituents into the reaction mixture leads to a bathochromic shift in all test samples. The value of bathochromic shift for extracts obtained by Method I is in the range of 65–85 nm and maxima are observed at 420–431 nm. The values of bathochromic shift for test samples obtained by methods II and III practically coincide and are slightly less than 65–80 nm, and the absorption maxima are in the wavelength range of 405–420 nm. The difference in the spectral behavior of flavonoids can be explained by the fact that flavones and flavonols from AlCl_3_, which contain hydroxyl groups at C-3 and/or C-5 and C-4 ketol groups, form acid-resistant complexes and acid-labile complexes with ortho-dihydroxyl systems (3′-OH and 4′-OH groups of ring B) [38,46]. The complexes formed between AlCl_3_ and the ortho-dihydroxyl groups of ring B decompose in the presence of acid. Thus, by performing the complexation reaction of flavonoids with AlCl_3_ in various reaction media under these experimental conditions, it can be assumed that there are such groups of flavonoids as flavones and flavonols with a potential antioxidant effect [49].

The TFC of *Rh. tomentosum* determined in liquid extracts under the conditions of three methods and expressed as equivalents of rutin, hyperoside, and quercetin are shown in Figure 5. As can be seen from the data, the use of quercetin as a standard substance to recalculate the total amount of flavonoids in *Rh. tomentosum* extracts under these experimental conditions can give both false-positive and false-negative results. The question of the dependence of the results of determining the total flavonoid content on the choice of the standard of flavonoids, experimental conditions, and selected wavelengths for calculations is of critical importance and is discussed by the authors in publications [43,44,45]. Thus, the question of the dependence of the results of determining the amount of flavonoids on the experiment conditions and the selected wavelengths for calculations is of critical importance and affects the final result of the determination.

For the raw material of *Rh. tomentosum* shoots, according to the results of chromatographic and spectrophotometric studies, it is advisable to use rutin or hyperoside as a standard substance. Regardless of the selected markers and method, the largest amount of flavonoids was found in the samples obtained using 60%, 70%, and 80% ethyl alcohol as an extractant.

Analyzing the antioxidant activity of the *Rh. tomentosum* extracts using the DPPH assay (Figure 6), it was observed that the extracts obtained by extraction with ethanol concentrations ranging from 30% to 90% exhibit slightly higher values of radical scavenging activity, falling within the range of 15.19 ± 1.14%–30.65 ± 6.75%. There is a correlation between the values obtained during the determination of TPC and antioxidant capacity. Quercetin was used as a standard antioxidant, positive control, and its calibration curve was determined in the range of concentration from 0.1 to 1.0 µg/mL. Quercetin equivalent antioxidant capacity was calculated using the regression equation between radical scavenging activity (%) of the extracts of *Rh. tomentosum* and mg QEAC/mL (Pearson’s *r* 0.996, *p* < 0.05). The quercetin equivalent antioxidant capacity of the extracts of *Rh. tomentosum* ranged from 0.88 ± 0.08 to 2.02 ± 0.49 mgQEAC/mL. However, no significant difference in the antioxidant activity of the samples was found, and the obtained values were within the standard deviation. 

**Figure 9:**
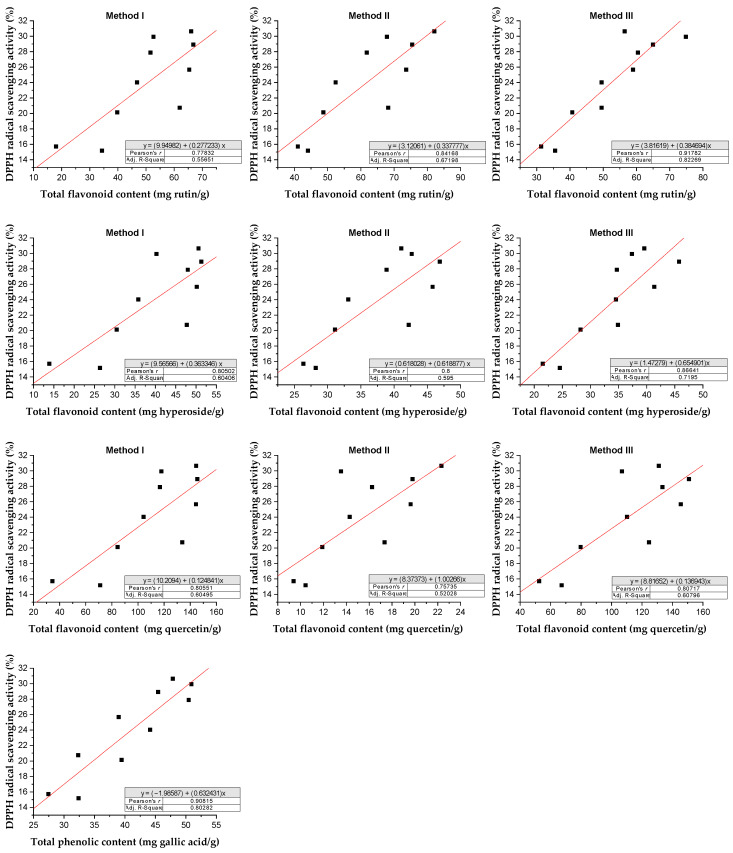
Relationship between total flavonoid content and total phenolic content and antioxidant activity of *Rh. tomentosum* by DPPH assay.

The relationship between total flavonoid content under different methods and total phenolic and antioxidant activity using the DPPH assay extract of *Rh. tomentosum* is shown in Figure 9. 

Following the DPPH assay, regression analysis shows that phenolic compounds contribute to about 80% (*r*^2^ = 0.8028, *p* < 0.05) of the radical scavenging properties in the extract of *Rh. tomentosum* [50,51]. Similarly, flavonoids contribute to about 55–82% (*r*^2^ = 0.5565, *p* < 0.05; *r*^2^ = 0.6719, *p* < 0.05; *r*^2^ = 0.8226, *p* < 0.05 under the methods I, II, and II, respectively) expressed as rutin equivalents, about 60–72% (*r*^2^ = 0.60406, *p* < 0.05; *r*^2^ = 0.595, *p* < 0.05; *r*^2^ = 0.7195, *p* < 0.05 under the methods I, II, and II, respectively) expressed as hyperoside equivalents, and about 52–61% (*r*^2^ = 0.6049, *p* < 0.05; *r*^2^ = 0.5202, *p* < 0.05; *r*^2^ = 0.6079, *p* < 0.05 under the methods I, II, and II, respectively) expressed as quercetin equivalents. Evidently, the rest of the proportion of antioxidant activity comes from nonphenolic compounds. Phenolics and flavonoids, in general, constitute a major group of compounds that act as primary antioxidants and are known to react with superoxide anion radicals, hydroxyl radicals, and lipid peroxyl radicals. They are also known to protect DNA from oxidative damage, inhibit the growth of tumor cells, and possess anti-inflammatory and antimicrobial properties [52,53].

When conducting microbiological studies, unexpected results were obtained, which require detailed further studies of the chemical composition of *Rh. tomentosum* extracts obtained during extraction with water and low-concentration ethanol. Despite the quantitative analysis showing a lower presence of polyphenols and flavonoids in extracts obtained with water and lower ethanol concentrations, ethanol extracts at 10%, 20%, and 30%, and especially at 60% and 70%, concentrations demonstrated significant antimicrobial effectiveness against both Gram-positive and Gram-negative bacteria, with water extracts also displaying this activity, albeit to a reduced degree. As can be seen from the data in Table 2, ethanol extract EtOH30 completely inhibits the growth of test cultures of microorganisms in 1:1 and 1:2 dilutions of the clinical strains #211 *Staphylococcus aureus* and #222 *Enterococcus* spp. and the reference strain *Pseudomonas aeruginosa* ATCC 10145. In a solid nutrient medium (agar diffusion method), extracts EtOH70 and EtOH 60 showed the best antimicrobial activity, but the presence of secondary growth of bacteria may indicate an insufficient amount of active substances in these extracts. On the other hand, water extract and EtOH30 did not show any effect on solid agar, in contrast to liquid medium, which may indicate poor diffusion of active substances.

## 4. Materials and Methods

### 4.1. Chemicals

All the chemicals and reagents used were of analytical grade. Ethanol, methanol, ethyl acetate, formic acid, ammonium acetate, glacial acetic acid, aluminum chloride hexahydrate, 2,2-Diphenyl-1-picrylhydrazyl (DPPH), Folin-Ciocalteu reagent, and sodium carbonate were purchased from Poch S.A. (Gliwice, Poland). The standards of rutin, hyperoside, quercetin, rosmarinic acid, caffeic acid, gallic acid, and chlorogenic acid were purchased from Sigma-Aldrich (Poznań, Poland). HPTLC analyses were performed on 20 cm × 10 cm HPTLC silica gel 60 F254 plates (Merck, Darmstadt, Germany).

### 4.2. Plant Material

The shoots of *Rh. tomentosum* (*Ledum palustre*) were collected at the fruiting stage in the forest belts of the Rivne region (Ukraine, 51.302381° N, 26.509154° E). The identity of the raw material was established by Nadiia Kovalska, Assoc. Prof. of the Department of Pharmacognosy and Botany of Bogomolets National Medical University. The voucher specimens of the plant have been deposited in the herbarium of the department [54,55,56]. The shoots were shade-dried at 25–35 °C and stored in tightly closed containers.

### 4.3. Extracts Preparation

Extracts from *Rh. tomentosum* shoots were obtained using a modified method that combined the effects of ultrasound and temperature to maximize the extraction of biologically active substances from the raw material [28,57,58,59]. Extracts were prepared using different concentrations of hydroalcoholic mixture and purified water to determine the effect of ethanol concentration on the extraction of flavonoids in the form of aglycones and glycosides since glycosidic and aglyconic forms of flavonoids have different solubility [27,60,61]. The use of ethanol as an extractant is the most acceptable in the pharmaceutical industry for the manufacture of herbal medicines and does not require a test for residual amounts of extractants in plant extracts. About 5.0 g of raw material (exact weight) was crushed to the size of particles passing through a 60 mesh sieve, placed in a flask with a thin section with a capacity of 100 mL, and 50 mL of the corresponding extractant was added to it. An extractant ethanol of various concentrations was used: 10%, 20%, 30%, 40%, 50%, 60%, 70%, 80%, 90%, or purified water. The flask was sealed and left to infuse for 24 h at room temperature (21 ± 1) °C. After infusion, ultrasonic extraction was performed by placing the flasks in an ultrasonic bath (Elmasonic S30H, Elma Schrnidbauer GmbH) for 30 min. Under the influence of ultrasound, there is an unauthorized increase in temperature, which at the end of the ultrasonic extraction of raw materials was (45 ± 0.5) °C. The resulting extract was filtered through cotton wool so that the particles of raw materials did not fall on the filter. Extraction was carried out twice more under the conditions described above with 25 mL of fresh extractant, filtering the extracts into the same flask. The first portion of the extract was collected separately, the second and third portions were combined and evaporated to dryness (rotary evaporator Heating Bath B-100, BUCHI) at a temperature of (45 ± 0.2) °C and a vacuum of 0.01 MPa; the resulting dry residue was dissolved in the first portion of the extract. Thus, the extracts to be studied were prepared in the ratio of raw material to extractant 1:5. 

### 4.4. Chromatographic Analyses

The test samples and standard substances were applied to the plates using an automatic HPTLC application device (Linomat 5, CAMAG, Muttenz, Switzerland). For chromatographic studies, extracts (2 mL) were additionally filtered through a Millipore filter with a pore size of 0.45 µm. The sample application volume was 5 µL. Methanol solutions of standard substances were prepared at the following concentrations: rutin 1 mg/mL, hyperoside 1 mg/mL, quercetin 0.2 mg/mL, rosmarinic acid 1 mg/mL, caffeic acid 1 mg/mL, and chlorogenic acid 0.2 mg/mL. 

Chromatographic separation was performed on HPTLC plates in a vertical glass chamber (CAMAG). In the mobile phase, the ethyl acetate/formic acid/water ratio was 15:1:1 [31,62,63]. A total of 70 mL of the mobile phase was used, and the chamber saturation time with the mobile phase was 40 min. After the eluent covered the distance from the start line to the finish line, the plates were removed from the chamber and dried in an oven at (105 ± 2) °C. Detection was based on natural fluorescence before and after derivatization by sequentially spraying 2-aminoethyl diphenylborate (10 g/L) and macrogol 400 (50 g/L) in UV light at 254 and 366 nm. The obtained chromatographic images were analyzed using HPTLC software (visionCATS, CAMAG).

### 4.5. Method for Determination of Total Phenolic Content

Total phenolic content (TPC) was determined by using the Folin-Ciocalteu method [64]. Briefly, 0.25 mL of the diluted sample was added to 0.25 mL 1:1 diluted Folin-Ciocalteu reagent, and then 0.5 mL of saturated sodium carbonate solution (200 g/L) was added after 4 min, along with 4.0 mL of water. After incubation at room temperature (23–25 °C) for 45 min, the absorbance of the mixture was measured at 760 nm using a U-3900/3900H Hitachi UV–Vis spectrophotometer (Hitachi, Tokyo, Japan). The compensation solution was water. All analyses were performed in triplicate. The total phenolic content was expressed as gallic acid equivalents (mg of GAE/g) according to the formula:

$$X,\;\mathrm{mg}/g=\frac{A\cdot m_{\mathrm{st}}\cdot K}{A_{\mathrm{st}}\cdot m}\cdot 1000,$$

where X—the total phenolic content in terms of gallic acid, mg/g;

K—dilution factor;

A—absorption of diluted sample at 760 nm;

A_st_—absorption of solutions of gallic acid at 760 nm.

The calibration range of gallic acid was from 5.45 to 12.48 µg/mL. 

### 4.6. Method for Determining the Total Flavonoid Content

The content of flavonoids was determined using differential spectrophotometry based on the formation of complexes of aluminum ions with flavonoids in a different reaction medium according to the method described in article [45]. Determination was carried out in methanol according to three methods that differed in the reaction medium—the first with the addition of only AlCl_3_, the second with AlCl_3_ and CH_3_COOH, and the third with AlCl_3_ and CH_3_COONH_4_ [44,65,66]. For the UV–Vis analysis, the original test samples were diluted with methanol so that the maximum absorption at the wavelengths of interest (λmax) was within the range of 0.6–0.8.

Method I: 2 mL of methanol, 0.5 mL of the test extract sample (or standard), and 0.20 mL of a 10% solution of AlCl_3_ in methanol were successively added to a 5.0 mL flask and held for 3 min; the volume of methanol was adjusted to 5.0 mL. 

Method II: 2 mL of methanol, 0.5 mL of the test extract sample (or standard). and 0.20 mL of a 10% solution of AlCl_3_ in methanol were successively added to a 5.0 mL flask and held for 3 min. Next, 0.2 mL of a 1M solution of glacial acetic acid in methanol was added. and the volume was adjusted to 5.0 mL with methanol. 

Method III: 2 mL of methanol, 0.5 mL of the test extract sample (or standard). and 0.20 mL of a 10% solution of AlCl_3_ in methanol were successively added to a 5.0 mL flask and held for 3 min. Next, 0.2 mL of a 1M solution of ammonium acetate in methanol was added. and the volume was adjusted to 5.0 mL with methanol. 

Compensation solutions were prepared similarly but without the addition of AlCl_3_.

The reaction mixtures were thoroughly stirred, held for 30 min at room temperature, and subjected to spectrophotometric analysis in the range of 280 to 500 nm (U-3900/3900H Hitachi). All analyses were performed in triplicate.

The total content of flavonoids in the studied extracts was calculated by the standard method in terms of rutin, hyperoside, or quercetin according to the formula as in the Section 4.5.

Where X—the total content of flavonoids in terms of rutin, hyperoside, or quercetin, mg/g;

K—dilution factor;

A—absorption at 437 nm (Method I), 409 nm (Method II), or 406 nm (Method III) for conversion to rutin; at 436 nm (Method I), 407 nm (Method II), or 407 nm (Method III) for conversion to hyperoside; at 456 nm (Method I), 434 nm (Method II), or 433 nm (Method III) for conversion to quercetin;

A_st_—absorption of solutions of standard samples of flavonoids at the same wavelengths. 

### 4.7. DPPH Radical Scavenging Activity

Antioxidant activities of the samples were analyzed by investigating their abilities to scavenge the DPPH% [67]. Briefly, a 76 µM solution of DPPH (1,1-diphenyl-2-picrylhydrazyl) was prepared using 96% ethanol. Then, 0.05 mL of the tested extract was added to 1.95 mL of the DPPH solution in 2 mL tubes. The blank consisted of the same volume of the extract and 1.95 mL of 96% ethanol. The mixtures were then mixed vigorously and allowed to stand at room temperature in the dark for 40 min. The absorbance of the resulting mixtures was read at a wavelength of 517 nm in 40 min, using the spectrophotometer U-3900/3900H Hitachi. The DPPH radical scavenging activity was computed according to the following equation:

$$\mathrm{DPPH}\;\mathrm{radical}\;\mathrm{scavenging}\;\mathrm{activity},\;\left(\%\right)=\frac{A_{\mathrm{control}\;}-A_{\mathrm{sample}}}{A_{\mathrm{control}}}\;\cdot 100$$

where A_control_ is the absorbance of the solution of DPPH against 96% ethanol;

A_sample_ is the absorbance of the reaction mixtures of the extract with DPPH at a wavelength of 517 nm against the same volume of the extract and 1.95 mL of 96% ethanol. 

The reaction mixture for measuring A_control_ consisted of 1.95 mL of 76 µM solution of DPPH and 0.05 mL of 96% ethanol. All analyses were performed in triplicate. Quercetin was used as a reference standard, and the results were expressed as mg quercetin/mL extract of *Rh. tomentosum*.

### 4.8. Antimicrobial Activity In Vitro

The studied extracts were tested for antimicrobial activity by the method of diffusion in agar and the method of serial dilutions [68,69,70]. For the agar diffusion method, 100 μL of the extract was sprinkled into a well in an agar plate (MPA, Sabouraud), where a bacterial suspension (McFarland 0.5) was inoculated. For the broth serial dilutions assay, we added 50 μL of meat-peptone broth and 100 μL of the studied extract to the first well, mixed, and 50 μL was transferred to the second well (second dilution), which contained 50 μL of the broth, and so on. Then, 50 μL of pure bacterial suspension (0.5 McFarland) was added to each well. The bacterial control contained 50 μL of broth + 50 μL of pure bacterial suspension. The plate was incubated for 24 h (37 °C). To count the number of colonies from each well, a sterile disposable loop (1 μL) was applied to a sector of an agar plate (MPA). After 24 h of incubation, colonies were counted on each sector. The control of test microorganisms was the total bacterial growth.

Ten reference and clinical microbial and fungal strains were used, previously identified by the MALDI TOF system (Bruker, Bremen, Germany) and 16S rRNA gene sequences. Reference strains: *Candida albicans* ATCC 885-653, *Staphylococcus aureus* ATCC 25923, *Pseudomonas aeruginosa* ATCC 10145, *Aspergilus niger*. All clinical strains (b2 *Staphylococcus aureus*, biofilm-forming; 223 *Staphylococcus delphinium*; 211 *Staphylococcus aureus*; 222 *Enterococcus* spp, 218 *Klebsiella pneumonia*, and 221 *E. coli*) were multidrug-resistant or extensively drug-resistant with different antibiotic resistance patterns. Clinical strains were isolated from a patient with healthcare-associated infections from regional hospitals. All testing was repeated in triplicate.

### 4.9. Statistical Analysis

The mean and standard deviation (SD) were calculated according to the monograph “Statistical Analysis of the Results of a Chemical Experiment” of the State Pharmacopoeia of Ukraine [71]. The average value was established based on 3 measurements. Values of the confidence interval were calculated using Student’s criterion limit. The data are presented as the mean ± SD [46].

## 5. Conclusions

Using an inexpensive, fast, and reproducible technique (HPTLC) and test markers, the presence of substances of flavonoid structure, rutin, hyperoside, and quercetin, and hydroxycinnamic acids, chlorogenic acid, were established in all the studied samples. The obtained results show that the HPTLC is the method of choice for the analysis of plant herbal extracts. The TPC and TFC were estimated using spectrophotometric methods involving the Folin-Ciocalteu reagent and the complexation reaction with aluminum chloride. The TPC expressed as gallic acid equivalents ranged from 27.42 ± 0.13 to 50.91 ± 2.78 mg GAE/g. Spectrophotometric techniques are proposed based on the reaction of the complexation of flavonoids with aluminum chloride using appropriate standard samples of flavonoid rutin, hyperoside, and quercetin. It has been proven that the course of the complexation reaction, according to the quantification of the flavonoid structure substances by the spectrophotometric method, is influenced by some factors, including the environment, the structure of the complexes, etc. Regardless of the chosen method, for the further production of *Rh. tomentosum* extracts with a higher content of flavonoid structure substance, it is advisable to use 60%, 70%, and 80% ethyl alcohol as the extractant. Following the DPPH assay, regression analysis shows that phenolic compounds contribute to about 80% (*r*^2^ = 0.8028, *p* < 0.05) of the radical scavenging properties in the extract of *Rh. tomentosum*. The microbiological evaluation demonstrated that ethanol extracts, particularly those with concentrations of 60% and 70%, as well as those with 10%, 20%, and 30%, were effective against both Gram-positive (clinical and reference staphylococci) and Gram-negative bacteria (reference Pseudomonas aeruginosa). The water-based extract also displayed antimicrobial properties, though its efficacy was comparatively lower. 

## Figures and Tables

**Table 1:** Summary of pharmacological studies of *Rh. tomentosum*.

Confirmed Biological Activity	Extract Type	Probable Active Compounds	Method Applied	Results	Source
Anticancer potential	Dried extracts obtained by extraction with water, 70% ethanol, isopropanol, acetone, and chloroform	May be attributed to ursolic acid as a constituent component	In vitro using primary human myeloid leukemia cell line patient samples, in vivro on mice engrafted with C1498 cells	This study demonstrated that extracts can exert anti-AML activity	[9]
Antidiabetic	Dried extracts obtained by extraction with 80% ethanol	Polyphenols	In vitro using the Caco-2 human enterocytic cell line and in vivo using an oral glucose tolerance test (OGTT)	These studies indicate a decrease in glucose absorption during an OGTT in normoglycemic rats and a significant decrease in the protein expression of SGLT1 and GLUT2 in CaCo2/15 cells	[10]
Antidiabetic potential	Dried extracts obtained by extraction with 80% ethanol	Chlorogenic acid, catechins, taxifolin, and quercetin glycosides	Using potentiation of basal and insulin-stimulated glucose uptake by skeletal muscle cells (C2C12) and adipocytes (3T3-L1), potentiation of glucose-stimulated insulin secretion by pancreatic b cells (bTC), potentiation of adipogenesis in 3T3-L1 cells, protection against glucose toxicity and glucose deprivation in PC12-AC neuronal precursor cells, and DPPH oxygen free radical scavenging	The present study revealed that *Rh. tomentosum* exhibits a promising profile of antidiabetic potential and is a good candidate for more in-depth evaluation	[11]
Antioxidant and anti-inflammatory activities	Extract obtained by extraction with 70% ethanol and was fractionated by EtOAc	Unknown	Antioxidant by DPPH and ABTS assay. The anti-inflammatory activities by the inhibitory activity against NO, PGE2, TNF-α, IL-1β, and IL-6 production on LPS-stimulated raw 264.7 macrophages	Extracts have high antioxidant activities similar to vitamin C, and the low concentration of extracts have high anti-inflammatory activities	[12]
Analgesic and anti-inflammatory activities	Essential oil, methanol extract, and aqueous extract	Flavonoid components	In vivo using model of acetic acid-induced writhing response and anti-inflammatory effect by using model of lambda-carrageenan-induced paw adema in mice	The analgesic and anti-inflammatory effects of methanol extract	[13]
Anti-inflammatory effect	Novogalene agent Ledum 50 obtained by 50% ethanol	Polyphenolic compounds	In vivo model of acute bronchitis initiated by endotracheal administration of 1% formalin solution	Normalization of the histostructure of the respiratory system	[14]
Anti-inflammatory activity	Dried extracts obtained by extraction with water	Unknown	In vitro using evaluation of inhibitory activity on prostaglandin biosynthesis and platelet-activating factor (PAF)-induced exocytosis	High inhibition was obtained, prostaglandin inhibition 50 ± 4%, PAF–exocytosis inhibition 71 ± 3%	[15]
Toxic, antioxidant, and antifungal activity	Essential oils obtained by hydrodistillation	The major compounds were palustrol, ledol, ascaridol isomers, myrcene, and cyclocolorenone isomers	The toxic activity using a brine shrimp (*Artemia* sp.) bioassay, DPPH and ABTS assay, and antifungal activity by agar disc diffusion assay (*Candida parapsilosis*)	Notable toxic activity of essential oils, high abilities to scavenge radicals, possess potential antifungal activity	[16]
Antioxidant and antimicrobial activities	Essential oil obtained by hydrodistillation and methanol extracts	The major compounds sabinene, terpinen-4-ol, myrtenal, α-pinen, β-selinene, α-selinene, and γ-elemene	Antioxidant activity by TBARS, NBT, and DPPH assay and inhibition of lipid peroxide formation. Antimicrobial activity by MIC (minimum inhibitory concentration) test and disc diffusion method	The methanol extract and essential oil showed antioxidant activity. The oil showed antimicrobial activity against *Streptococcus pneumoniae*, *Clostridium perfringens*, *Candida albicans*, *Mycobacterium smegmatis*, *Acinetobacter lwoffii*, and *Candida krusei*, while the methanolic extracts exhibited slight or no activity	[17]
Antioxidant activities	Essential oil obtained by hydrodistillation, extract obtained by extraction with 70% ethanol	The major compounds 4-thujene, α-thujenal, and (-)-4-terpineolwhile. In the ethanol extract were α-farnesene, n-tetradecane, hexadecanoic acid, oblivon c, and lilac aldehyde	DPPH and ABTS assay	Ethanol extract showed stronger antioxidant activities than the essential oil	[18]
Antimicrobial activities	Essential oil obtained by steam distillation, CO_2_ by supercritical carbon dioxide	The main compounds EO palustrol, myrcene, and ledol. In scCO2 extract, the major components palustrol and ledol, and myrcene absent	Using modified agar well diffusion methods *against S. aureus*, *P. aeruginosa*, *C. albicans*, *A. niger*, *C. cladosporioides*, and *P. venetum*	All the EOs and scCO2 extracts showed a broad spectrum of antimicrobial activities against the selected microbes	[19]
Antibacterial activity	Essential oil was isolated by subcritical fluid extraction technology with butane as a solvent	The main constituents α-thujenal, bicyclocompounds, β-phellandrene, benzene,1-methyl-3-(1-methylethyl, propanal,2-methyl-3-phenyl, and β-terpineol	Using the modified disc method	Extract expressed good antibacterial activity against the marine pathogen *V. parahaemolyticus*	[20]
Insecticidal activity on mosquitoes, moths, and flies	Essential oil obtained by hydrodistillation	The main constituents p-cymene, isoascaridole, and cis-ascaridole	Culex quinquefasciatus larvae, *M. domestica* adults, *S. littoralis* early 3rd instar larvae. Toxicity on the nontarget aquatic microcrustacean *Daphnia magna* and soil organisms *Eisenia fetida*. Toxicity keratinocytes cell line *(HaCaT)*, primary human fibroblast cell line (NHF A12), and MTT assay	Essential oil showed significant mortality on the larvae of C. quinquefasciatus and *S. littoralis* and adults of *M. domestica*, with little or no impact on beneficial organisms such as aquatic microcrustacean and earthworms and moderate toxicity on human fibroblasts and keratinocytes	[21]
Mosquito repellent	Volatile compounds were collected by solid phase microextraction, essential oil was obtained by steam distillation, extracts were extracted with hexane, ethyl acetate, or methanol	The volatile fraction of an ethyl acetate extract has the major compounds p-cymene, terpinyl acetate, sabinene, p-pinene, bornyl acetate, α pinene, β-phellandrene, camphene, Z-ocimene, and γ-terpinene	Repellency bioassays were carried out using cages made of mosquito netting and field experiment	Ethyl acetate extracts significantly reduced the probing activity of *Aedes aegypti* (L.) and reduced biting by mosquitoes	[22]
Repellency activity	Oil, 10%, diluted in acetone	Compounds of essential oil	A repellency bioassay on *I. ricinus* nymphs	Exhibited 95% repellency	[23]
Antihyperuricmic	Mother tincture *Ledum palustre* L., potency 30c and 1M	Homeopathy	Potassium oxonate induced rat model	The present study indicated marked hypouricemic effects; however, clear conclusion of hypouricemic potential of *Ledum palustre* required replication of experiment	[24]
Antihyperuricmic	Mother tincture *Ledum palustre* L.	Homeopathy	Randomized single-blind experimental design was applied to the study (200 humans). For analysis of uric acid in blood samples, the enzymatic method was selected	The value of reduction in serum uric acid in males was 4.3 ± 0.3, in females was 4.6 ± 0.4	[25]

**Table 2:** Antimicrobial effect of *Rh. tomentosum* extracts by the method of serial dilutions (1:1; 1:2), number of colony-forming units (CFU).

	№195 *Staphylococcus aureus* ATCC 25923		№ b2 *Staphylococcus aureus* Biofilm-Forming		№ 223 *Staphylococcus delphinium*		№211 *Staphylococcus aureus*		№222 *Enterococcus* spp.		192 *Pseudomonas aeruginosa* ATCC 10145	
Dilution	1:1	1:2	1:1	1:2	1:1	1:2	1:1	1:2	1:1	1:2	1:1	1:2
EtOH0	80	TG	10	10^5^	50	10^5^	30	150	45	10^5^	2	50
EtOH10	1	60	80	160	0	80	0	80	11	0	90	TG
EtOH20	0	45	0	200	11	5	0	1	0	0	0	TG
EtOH30	0	0	0	40	0	4	0	3	0	0	0	90
EtOH40	0	0	0	0	0	0	0	0	0	0	0	0
EtOH50	0	0	0	0	0	0	0	0	0	0	0	0
EtOH60	0	0	0	2	0	0	0	0	0	0	0	0
EtOH70	0	0	0	0	0	0	0	0	0	0	0	0
EtOH80	0	0	0	0	0	0	0	0	0	0	0	0
EtOH90	0	0	0	0	0	0	0	0	0	0	0	0

0—no growth; CFU—colony-forming units; TG—total solid growth.

## Data Availability

The data presented in this study are available on request from the corresponding author.
